# Randomized, Double-Blind, Placebo-Controlled, Clinical Study of Passiflora incarnata in Participants With Stress and Sleep Problems

**DOI:** 10.7759/cureus.56530

**Published:** 2024-03-20

**Authors:** Mahesh Kumar Harit, Narendra Mundhe, Sanjay Tamoli, Vinay Pawar, Vedvati Bhapkar, Ganesh Kolhe, Swapnali Mahadik, Anand Kulkarni, Ankit Agarwal

**Affiliations:** 1 Department of Sanskrit Samhita Siddhant, DY (Dnyandeo Yashwantrao) Patil Deemed to be University School of Ayurveda, Navi Mumbai, IND; 2 Department of Kayachikitsa, KVTR (Karmvir Vyankatrao Tanaji Randhir) Ayurved College, Dhule, IND; 3 Department of Research, Target Institute of Medical Education and Research, Mumbai, IND; 4 Department of Rasashastra and Bhaishajya Kalpana, DY (Dnyandeo Yashwantrao) Patil Deemed to be University School of Ayurveda, Navi Mumbai, IND; 5 Department of Research, JK (Jairamdass Khushiram) Botanicals Private Limited, Navi Mumbai, IND

**Keywords:** passion flower, sleep, stress, clinical study, sivi (passiflora incarnata extract)

## Abstract

Background and objectives

SIVI is a standardized extract prepared using the aerial parts of *Passiflora incarnata* developed to enhance the quality of sleep. ​​​​​​The objective of the present study was to the evaluate efficacy and safety of SIVI (*Passiflora incarnata* extract) in the management of stress and sleep problems in Indian participants in a randomized, double-blind, placebo-controlled, clinical study.

Materials and methods

A total of 65 participants with stress and insomnia were randomized to two groups with 32 in the SIVI (*Passiflora incarnata* extract) group and 33 in the placebo group. Subjects were asked to take the test substance along with water at bedtime for 30 days. The Perceived Stress Scale, quality of life using the General Health Questionnaire (GHQ-12) scale, and Insomnia Severity Index were assessed on day 1, day 15, and day 30.

Results

*Passiflora* *incarnata* extract showed a statistically significant reduction in the mean score of stress on the Perceived Stress Scale and significantly increased the mean score of total sleep time compared to placebo. The general psychological health was found to be significantly improved in the SIVI (*Passiflora* *incarnata* extract) group compared to the placebo group on day 15 and day 30. SIVI (*Passiflora incarnata* extract) did not show any adverse effects.

Conclusions

The results of the current study indicate that *Passiflora incarnata* extract is beneficial in the management of stress and helps to improve sleep quality in subjects with stress and insomnia.

## Introduction

Stress is the physical, emotional, or intellectual strain caused because of a response to what happens around us. In humans, stress typically describes a negative or a positive condition that can have an impact on a person's mental and physical well-being [1]. Stress can increase the risk of strokes, heart attacks, ulcers, and mental illnesses such as depression [2]. Excess stress can manifest itself in a variety of emotional, behavioral, and even physical symptoms. Symptoms of stress vary extremely among different individuals. Common physical symptoms of excess stress include sleep disturbances or changes in sleeping habits (insomnia or excessive sleep), muscle tension, muscle aches, headaches, gastrointestinal problems, and fatigue [3]. Insomnia or sleeplessness is a sleep disorder, where people have trouble sleeping [4]. They may have difficulty either falling asleep or staying asleep as long as desired [5-7]. Insomnia is typically followed by low energy, daytime sleepiness, irritability, and a depressed mood. Insomnia may be short-term, lasting for days or weeks, or long-term, lasting more than a month [3]. Insomnia can occur independently or because of secondary effects of other conditions. Conditions, that cause insomnia, include psychological stress, chronic pain, hyperthyroidism, heartburn, restless leg syndrome, menopause, certain medications, and drugs such as caffeine, nicotine, and alcohol [8,9]. Other risk factors include working night shifts and sleep apnea [5]. Diagnosis is based on sleep habits and an examination to look for underlying causes [3]. People over the age of 65 years are more affected than younger people [8]. Females are more often affected than males [7]. Lifestyle changes and sleep hygiene are typically the first treatment for stress and sleep problems [10]. An important step in stress management and treatment of stress-related symptoms is exercise [11]. Other measures include cognitive behavioral therapy (CBT), which helps us to understand our thought patterns, recognize trigger points, and identify positive actions we can take, meditation, and yoga with a particular focus on reducing stress [12]. Tranquilizers and antidepressants can help to reduce or manage some of the signs of stress and insomnia. However, these drugs may cause nausea, increased appetite and weight gain, fatigue and drowsiness, dry mouth, blurred vision, and constipation [13].

*Passiflora incarnata* is used for treating anxiety or nervousness, generalized anxiety disorder (GAD), insomnia, neuralgia, convulsion, spasmodic asthma, palpitations, cardiac rhythm abnormalities, hypertension, etc. It has shown a calming effect, which helps to reduce stress and can therefore be helpful in the treatment of insomnia, anxiety, and depression. Research studies indicate that passion flower has a positive effect on sleep patterns. Passion flower reduces the time to fall asleep and increases the duration of sleep [14]. Looking at the activities of *Passiflora incarnata* (aerial parts) extract, a hypothesis was postulated that it would be useful in the management of stress and insomnia. To test the hypothesis, a clinical study titled "A randomized, double-blind, placebo-controlled, multi-centric, interventional, prospective clinical study to evaluate efficacy and safety of SIVI (*Passiflora incarnata* extract) in participants with stress and sleep problems" was carried out.

## Materials and methods

Design

The study was designed as a randomized, double-blind, placebo-controlled, clinical study and was conducted at two clinical sites in India, viz., KVTR (Karmvir Vyankatrao Tanaji Randhir) Ayurved College, Dhule, India, and DY (Dnyandeo Yashwantrao) Patil Deemed to be University School of Ayurveda, Navi Mumbai, India. The study was approved by the respective institutional ethics committees of the study centers (approval numbers: AMB/424/2021-22 and DYPUSA/22/186), and the study was registered in the Clinical Trials Registry - India (CTRI) on July 6, 2022, with the registration number CTRI/2022/07/043753. Study participants were divided into two groups, viz., the SIVI (*Passiflora incarnata* extract) group and the placebo group, in a ratio of 1:1. The SIVI (*Passiflora incarnata* extract) group was administered with the test substance (SIVI capsules), and the placebo group was administered with the placebo capsules. The total duration of treatment was one month (30 days). There were four visits planned for the subjects during the study, which included a visit for screening and a visit on day 0, day 15, and day 30. Primary outcomes and secondary outcomes were planned on all the visits.

Participants

Subjects attending the outpatient department of the study centers were considered for the study. Literate male and female subjects aged 18-60 years, subjects who perceive themselves as stressed and have a score between 14 and 24 on the Perceived Stress Scale (PSS), subjects with a score of 7-21 on the Insomnia Severity Index (ISI), and subjects who sign the informed consent form and are willing to follow the procedures specified the study protocol were included in the study. Subjects suffering from any known chronic physical, hormonal, or psychiatric illness, subjects using oral or systemic contraceptive medications, subjects with uncontrolled diabetes and hypertension, subjects with substance dependence (i.e., taking prohibited medications like opium, cannabis methamphetamines, etc.), chronic alcoholics, and habitual tobacco chewers, known cases of severe/chronic hepatic or renal disease, known subjects of any active malignancy, subjects with a known history of significant cardiovascular events in less than 12 weeks prior to recruitment, subjects having known chronic, contagious infectious diseases, such as active tuberculosis, hepatitis B or C, or HIV, known cases of active metabolic or gastrointestinal diseases that may interfere with nutrient absorption, metabolism, or excretion, excluding diabetes, subjects using any other investigational study product within one month prior to recruitment or subjects currently participating in any other clinical study, subjects with known hypersensitivity to any of the ingredients used in study products, and pregnant and lactating females were excluded from the study. Other conditions, such as known chronic skin diseases, psychosomatic diseases, neurological disorders, etc., which, in the opinion of the investigators, made subjects unsuitable for enrolment or could interfere with his/her participation in and completion of the study were also excluded from the study.

Interventions, sample size, and randomization

Capsules containing SIVI (*Passiflora incarnata* extract) prepared using the aerial parts of *Passiflora incarnata* were used as the intervention in the SIVI (*Passiflora incarnata* extract) group. A daily dose of 600 mg *Passiflora incarnata* extract was taken along with water by the subjects in the SIVI (*Passiflora incarnata* extract) group at bedtime for 30 days. Placebo capsules looking like SIVI (*Passiflora incarnata*) capsules were made using microcrystalline cellulose and were asked to be taken at bedtime by the placebo group. No formal sample size calculation was carried out as this being the first pilot study and a sample size of at least 30 subjects per group was considered sufficient by the investigators. A total of 65 participants were screened in the study, and all of them were recruited as there were no screen failures and randomized into two groups in a ratio of 1:1 using a computer-generated list.

Primary outcomes

Changes in stress and changes in the subject-reported total sleep time were used as the primary outcomes of the study. Stress was assessed using the PSS on day 0, day 15, and day 30. PSS scores were obtained by reversing responses (e.g., 0=4, 1=3, 2=2, 3=1, and 4=0) to the four positively stated items (items 4, 5, 7, and 8) and then summing across all scale items. A daily diary card was given to the subjects, and they were asked to record their total sleep time. Subject-reported total sleep time was noted from the respective subjects' diary on day 0, day 15, and day 30. Change in the stress and change in the total sleep time from the baseline to the end of the study in the SIVI (*Passiflora incarnata* extract) group was compared to the change in the stress and change in the total sleep time from the baseline to the end of the study in the placebo group. 

Secondary outcomes

Change in the general psychological health assessed using a short General Health Questionnaire (GHQ-12), subject-reported sleep onset, subject-reported number of awakenings, subject-reported wake time after sleep onset (WASO), sleep efficiency, the severity of insomnia using ISI, serum cortisol, daytime fatigue assessed using Fatigue Severity Scale (FSS), daytime mood, ability to function at work, concentration and memory on a graded scale, quality of sleep using Pittsburgh Sleep Quality Index (PSQI), and usage of rescue medications from the baseline to the end of the study and between the groups were considered as the secondary outcomes of the study. Subject-reported parameters were taken from the subjects' diary. All the secondary outcomes were carried out on days 0, 15, and 30 unless otherwise specified. Adverse events, vitals (blood pressure, pulse rate, respiration rate, and body temperature), safety assessments (complete blood count, liver function tests, renal function tests, lipid profile, and fasting blood sugar levels), and tolerability to the test substance were also assessed as part of the secondary outcomes.

The GHQ-12 consisted of 12 items, each assessing the severity of a mental problem over the past few weeks using a 4-point scale (from 0 to 3). The score was used to generate a total score ranging from 0 to 36, with higher scores indicating worse conditions. Sleep efficiency is defined as total sleep time divided by the time in bed multiplied by 100. The WASO is defined as the total awakening time from falling asleep to final awakening and was subjectively determined based on the subjects' diary. The ISI has seven questions. The seven answers are added up to get a total score. The last two weeks' severity of the insomnia problem was evaluated using this index. The severity of insomnia is graded based on the index as "no clinically significant insomnia" if the index is between 0 and 7, "sub-threshold insomnia" if the index is between 8 and 14, "clinical insomnia (moderate severity)" if the index is between 15 and 21, and "clinical insomnia (severe)" if the index is between 22 and 28. The FSS is a method of evaluating the impact of fatigue. The FSS questionnaire contains nine statements that rate the severity of fatigue. Each question is given a number from 1 to 7; the subject needs to circle the number that reflects his/her condition during the past week and the extent to which he/she agrees or disagrees that the statement applies to him/her. Subjects were allowed to use sedatives as rescue medication during the study period if required. However, they were asked to record the number of sedatives along with the dosage used in the case record form. The number of subjects who required rescue medication (sedatives) was calculated at the end of the study. Subjects were evaluated to check whether sleep problem interferes with his/her daily functioning such as daytime mood, ability to function at work, concentration, and memory on a graded scale (0=not at all interfering, 1=a little, 2=somewhat, 3=much, 4=very much interfering). The PSQI contains 19 self-rated questions and five questions rated by the bed partner or roommate. Only self-rated questions are included in the scoring. The 19 self-rated items are combined to form seven component scores, each of which has a range of 0-3 points. In all cases, a score of "0" indicates "no difficulty," while a score of 3 indicates "severe difficulty." The seven component scores are then added to yield one global score with a range of 0-21 points with "0" indicating "no difficulty" and "21" indicating severe difficulties in all areas. 

Adverse events and safety assessments

Evaluation of adverse events/adverse drug reactions at every follow-up visit and establishment of their relationship with the study product was assessed. The tolerability of the study product was evaluated by following the safety grades as "1" for excellent overall safety (no adverse event(s) reported), "2" for good overall safety (mild adverse event(s) reported which subside with or without medication), "3" for fair overall safety (moderate to severe adverse event(s) reported which subside with or without medication and do not necessitate stoppage of study products), and "4" for poor overall safety (serious adverse event(s) which necessitate stoppage of study). Safety was assessed by clinical review of all safety parameters, including the reported adverse events if any, vital signs including allergic reactions, etc., and parameters such as CBC, liver function tests, renal function tests, lipid profile, and fasting blood sugar.

Statistical methods

The data obtained in the studies were subjected to tests of significance using appropriate statistics. The data on discrete variables are represented as actual frequencies, i.e., n (%). The data on continuous variables are represented as mean±SD. GraphPad InStat Version 3.6 software was used for statistical analysis of data. A p-value of <0.05 was considered significant. Subjects who completed the study as per the protocol were considered as "per-protocol population" and subjects who took at least one dose of the study drug were considered as "safety population" and were evaluated accordingly.

## Results

Participant flow

**Figure 1:**
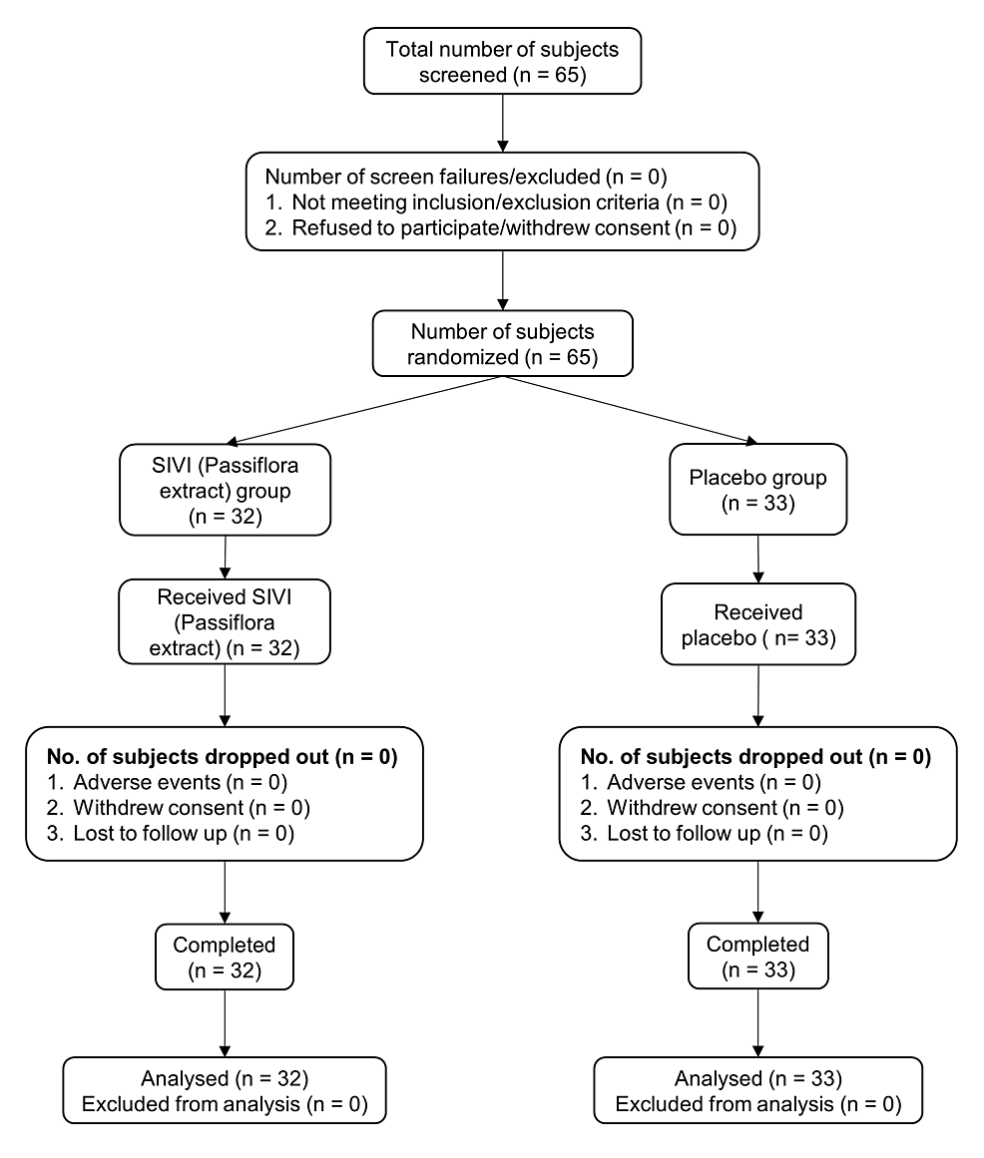
Flow of participants in the study (CONSORT flowchart) CONSORT: Consolidated Standards of Reporting Trials

A total of 65 participants were screened in the study, and all of them were found eligible as per the specified inclusion and exclusion criteria. Therefore, they were recruited for the study after obtaining the signed consent from each subject. All the recruited subjects were randomized into two groups (32 in the SIVI (*Passiflora incarnata* extract) group and 33 in the placebo group). There were no dropouts, and all 65 subjects completed the study (32 in the SIVI (*Passiflora incarnata* extract) group and 33 in the placebo group). Details of the participant's flow in the study are shown in Figure 1.

Demographic details of the subjects

The average age of the subjects was 38.63±11.86 years in the SIVI (*Passiflora incarnata* extract) group and 40.06±11.81 years in the placebo group. The number of males and females participating in the SIVI (*Passiflora incarnata* extract) group was 19 and 13, respectively, while the same in the placebo group was 15 and 18, respectively. There was no statistically significant difference seen in the demographic characteristics between the groups in the age and the gender.

Effect of SIVI (*Passiflora incarnata* extract) on stress

**Figure 2:**
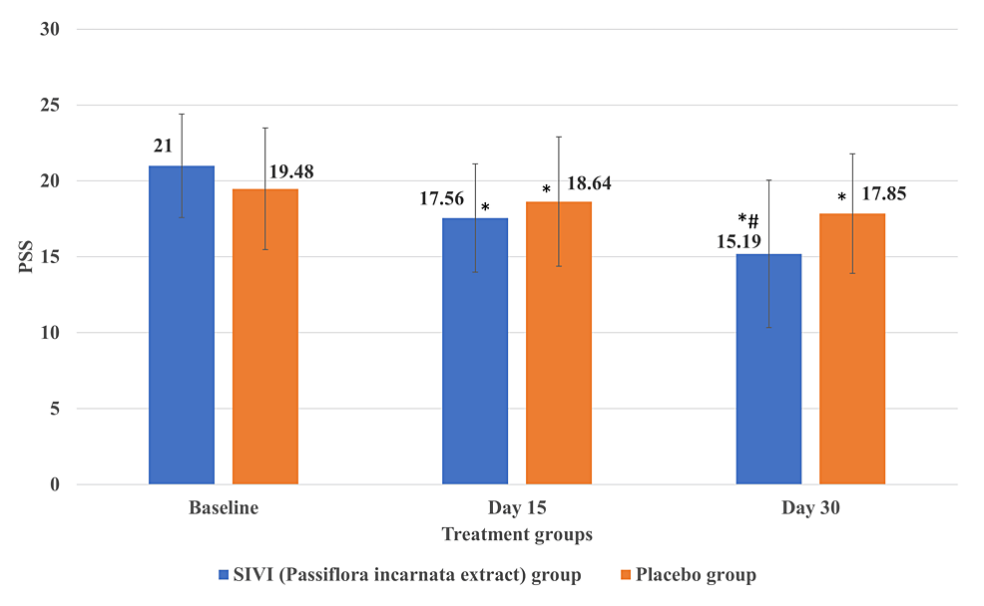
Effect of SIVI (Passiflora incarnata extract) on stress assessed using PSS Each value represents the mean±SD; n=32 for the *Passiflora *group and n=33 for the placebo group; *p<0.05 compared to the respective baseline; #p<0.05 compared to the placebo group. PSS: Perceived Stress Scale

As all the subjects completed the study, the numbers analyzed are equal to the number of subjects who participated in the study. The stress of subjects assessed using PSS in the SIVI (*Passiflora incarnata* extract) group was reduced significantly (p<0.05) on day 30 compared to the placebo group (Figure 2). There was a significant (p<0.05) reduction in the stress of subjects in both groups on day 15 and day 30 compared to the respective baseline stress levels.

Effect of SIVI (*Passiflora incarnata* extract) on total sleep time

**Figure 3:**
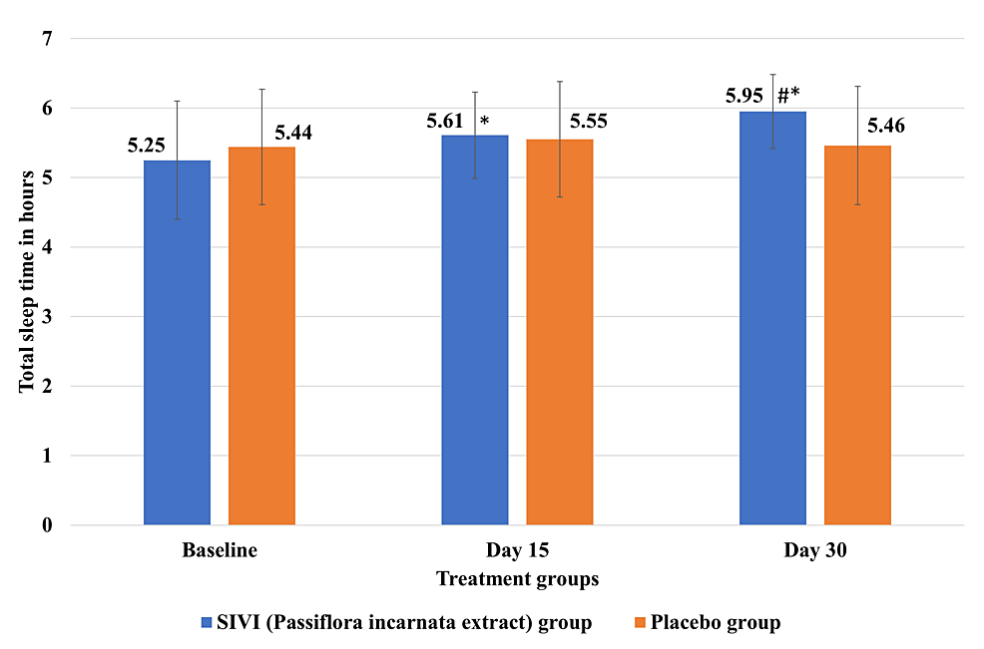
Effect of SIVI (Passiflora incarnata extract) on total sleep time Each value represents the mean±SD; n=32 for the *Passiflora *group and n=33 for the placebo group; *p<0.05 compared to the respective baseline; #p<0.05 compared to the placebo group.

SIVI (*Passiflora incarnata* extract) significantly (p<0.05) increased the mean total sleep time on day 30 compared to the placebo group (Figure 3). SIVI (*Passiflora incarnata* extract) showed a significant (p<0.05) increase in the mean total sleep time of subjects on day 15 and day 30 compared to its baseline, but the mean total sleep time was not significantly different on day 15 and day 30 in the placebo group compared to its baseline level.

Effect of SIVI (*Passiflora incarnata* extract) on other efficacy parameters

The results of the secondary outcome parameters are shown in Table 1. The general psychological health of subjects in the SIVI (*Passiflora incarnata* extract) group as assessed using GHQ-12 was found to be significantly (p<0.05) improved on day 15 and day 30 compared to the placebo group. The general psychological health of subjects in both groups showed significant improvement on day 15 and day 30 compared to their baseline. Sleep efficiency was found to be increased significantly (p<0.05) from the baseline level of 75.53% to 81.51% and 86.61% on day 15 and day 30, respectively, in the SIVI (*Passiflora incarnata* extract) group compared to the placebo group, which showed a negligible increase from the baseline level of 79.28% to 81.50% and 81.98% on day 15 and day 30, respectively. Subjects in the SIVI (*Passiflora incarnata* extract) group had significantly (p<0.05) lesser "time to sleep onset" on day 30 compared to the placebo group. Both groups showed significantly lesser "time to sleep onset" within their groups on day 15 and day 30 compared to their respective baseline.

**Table 1:** Effect of SIVI (Passiflora incarnata extract) on the secondary outcome parameters Each value represents the mean±SD; n=32 for the SIVI (*Passiflora incarnata* extract) group and n=33 for the placebo group; *p<0.05 compared to the respective baseline; #p<0.05 compared to the placebo group. ND: not determined; GHQ-12: General Health Questionnaire; WASO: wake time after sleep onset; ISI: Insomnia Severity Index; FSS: Fatigue Severity Scale; PSQI: Pittsburgh Sleep Quality Index

Secondary outcomes	SIVI (*Passiflora incarnata* extract) group			Placebo group		
	Baseline	Day 15	Day 30	Baseline	Day 15	Day 30
GHQ-12 (score)	24.13±3.92	17.53±2.27*#	15.47±3.51*#	22.27±3.98	21.03±4.13*	20.42±4.35*
Time to sleep onset (minutes)	72.19±13.97	53.91±20.11*	42.50±25.21*#	60.15±16.79	56.06±18.15*	52.88±21.33*
Number of awakenings	1.69±0.69	1.25±0.44*	0.56±0.72*#	1.24±0.44	1.21±0.42	1.00±0.75
WASO (minutes)	30.47±14.28	21.09±11.96*	10.94±14.28*#	27.58±14.74	25.00±15.36	20.76±19.45*
ISI (score)	16.25±2.93	12.40±2.51*#	10.63±2.94*#	17.18±3.25	16.21±3.53*	15.79±3.87*
Serum cortisol (µg/dL)	10.57±3.35	ND	8.75±3.22*	10.14±4.19	ND	9.02±3.44*
FSS (score)	43.22±7.94	31.91±7.32*	25.94±9.86*#	40.12±7.77	36.58±9.42*	36.00±9.96*
Interference of sleep on daytime mood (score)	3.22±0.42	2.19±0.47*#	1.50±0.84*#	3.09±0.29	2.64±0.60*	2.48±0.83*
Interference of sleep on ability to function at work (score)	3.22±0.42	2.19±0.47*#	1.50±0.84*#	3.00±0.00	2.70±0.64*	2.58±0.83*
Interference of sleep on concentration (score)	2.97±0.18	2.19±0.47*#	1.50±0.84*#	3.00±0.25	2.70±0.64*	2.58±0.83*
Interference of sleep on memory (score)	2.97±0.18	2.19±0.47*#	1.50±0.84*#	2.97±0.17	2.64±0.60*	2.48±0.83*
PSQI (score)	14.72±5.02	13.47±5.22*	9.00±5.1188*#	15.09±4.33	14.45±5.06	13.15±4.15*

"Number of awakenings" was found significantly lesser in the SIVI (*Passiflora incarnata* extract) group on day 30 compared to the placebo group. Subjects in the SIVI (*Passiflora incarnata* extract) group showed significant differences in the "number of awakenings" within the group on day 15 and day 30 compared to its baseline. However, subjects in the placebo group did not show any significant difference in the "number of awakenings" within the group on day 15 and day 30 compared to its baseline. Subjects in the *Passiflora* group had significantly (p<0.05) lower "WASO" on day 30 compared to the placebo group. The SIVI (*Passiflora incarnata* extract) group showed significantly lower "WASO" within the group on day 15 and day 30 compared to its baseline. However, the placebo group showed significantly lower "WASO" only on day 30 compared to its baseline.

The severity of insomnia in the SIVI (*Passiflora incarnata* extract) group as assessed using ISI was found to be significantly (p<0.05) reduced on day 15 and day 30 compared to the placebo group. Both groups showed significant improvement in the ISI within the group on day 15 and day 30 compared to their respective baseline. "Daytime fatigue" as assessed using FSS was significantly (p<0.05) lower on day 30 compared to the placebo group. Both groups showed significantly lesser FSS within the group on day 15 and day 30 compared to their respective baseline. Quality of sleep as assessed using PSQI was found to be significantly (p<0.05) improved in the SIVI (*Passiflora incarnata* extract) group on day 30 compared to the placebo. Quality of sleep was also found to be improved within the SIVI (*Passiflora incarnata* extract) group on day 15 and day 30 compared to its baseline. However, the quality of sleep was found to have improved in the placebo group only on day 30 compared to its baseline. None of the subjects in both groups required rescue medication (sedatives) during the study period.

Significant reduction in the serum cortisol was found on day 30 within the SIVI (*Passiflora incarnata* extract) and placebo groups compared to their respective baseline levels, but no significant difference was seen between the groups. Interference of sleep on daytime mood, ability to function at work, concentration, and memory were significantly (p<0.05) lesser in the SIVI (*Passiflora incarnata* extract) group on day 15 and day 30 compared to that of the placebo group and significantly lesser within each group on day 15 and day 30 compared to their respective baseline level.

Safety of SIVI (*Passiflora incarnata* extract)

SIVI (*Passiflora incarnata* extract) was found to be safe without any safety concerns compared to the placebo group. The pulse rate, respiratory rate, body temperature, systolic blood pressure, and diastolic blood pressure were within the normal range in both groups at the baseline and after treatment.

Results of the safety-related laboratory parameters including complete blood count, liver function tests, and renal function tests (Table 2) were in the normal range and showed no significant change from baseline within the group and between the groups.

**Table 2:** Results of the safety assessment parameters Each value represents the mean±SD; n=32 for the SIVI (*Passiflora incarnata* extract) group and n=33 for the placebo group. RBC: red blood cell; WBC: white blood cell; ESR: erythrocyte sedimentation rate; AST: aspartate aminotransferase; ALT: alanine aminotransferase; ALP: alkaline phosphatase; BUN: blood urea nitrogen

Safety parameters	SIVI (*Passiflora incarnata* extract) group		Placebo group	
	Baseline	Day 30	Baseline	Day 30
RBC (×10^6^ cells/µL)	4.31±0.56	4.32±0.57	4.36±0.49	4.44±0.52
WBC (cells/mm^3^)	6381.25±1839.6	6278.13±1371.5	6824.24±1620.8	6318.18±1002.3
Haemoglobin (g/dL)	12.79±2.13	12.72±2.06	12.68±1.69	12.71±1.53
Platelets (×10^5 ^cells/mm^3^)	2.73±0.76	2.63±0.71	2.79±0.58	2.82±0.68
ESR (mm/hour)	17.94±12.02	15.06±12.07	21.12±18.54	18.12±15.47
Total bilirubin (mg/dL)	0.77±0.23	0.74±0.22	0.75±0.26	0.74±0.23
Direct (mg/dL)	0.25±0.07	0.24±0.09	0.24±0.11	0.26±0.11
Indirect (mg/dL)	0.52±0.17	0.50±0.17	0.51±0.20	0.48±0.18
AST (U/L)	19.22±6.07	21.27±6.29	21.06±5.69	20.02±5.42
ALT (U/L)	28.79±12.46	28.82±10.62	30.81±10.54	28.24±11.37
ALP (U/L)	104.37±36.28	99.66±31.07	105.50±42.20	102.89±36.20
Total proteins (g/dL)	7.06±0.31	7.04±0.37	7.07±0.32	7.03±0.40
Serum albumin (g/dL)	4.04±0.45	4.05±0.35	3.91±0.50	3.93±0.34
Serum creatinine (mg/dL)	0.98±0.17	0.95±0.16	0.94±0.15	0.91±0.14
BUN (mg/dL)	10.33±2.34	10.53±2.77	10.08±2.52	9.93±2.68
Fasting blood sugar (mg/dL)	85.36±13.00	86.58±11.07	93.00±25.04	88.63±15.57

Adverse events

None of the subjects in the placebo group as well as in the SIVI (*Passiflora incarnata* extract) group reported any adverse events during the study indicating the safety and tolerability of the tested SIVI (*Passiflora incarnata* extract).

## Discussion

This article was previously posted to the Research Square preprint server on May 25, 2023. The study was conducted to evaluate the efficacy and safety of SIVI (*Passiflora incarnata* extract) in participants with stress and insomnia. A statistically significant reduction in the mean score of stress assessed using PSS was observed from the baseline visit to all follow-up visits in both groups. When compared between the groups, a statistically significant reduction in stress was observed in the *Passiflora* group compared to the placebo group on day 30. The mean score of total sleep time derived from the subject diary increased significantly from the baseline visit to every follow-up visit till the end of the study; however, no significant increase in total sleep time was observed till the end of the study in the placebo group. When compared between the groups, a statistically significant increase in total sleep time was observed in the *Passiflora* group compared to the placebo group on day 15 and day 30. The general psychological health assessed using a short General Health Questionnaire (GHQ-12) significantly improved in the *Passiflora *group compared to the placebo group on day 15 and day 30.

Patient-reported time to sleep onset was significantly reduced in the *Passiflora *group compared to the placebo group on day 15 and day 30. In the *Passiflora *group, the mean score of the patient-reported total number of awakenings as per the subject's diary significantly reduced on day 15 and day 30; however, in the placebo group, the reduction in the patient-reported total number of awakenings was non-significant on day 15 and day 30. In both groups, the mean score of patient-reported wake time after sleep onset (based on the subject's diary) was found significantly reduced on day 15 and day 30. When compared between the groups, a statistically significant reduction in the wake time after sleep onset (based on the subject's diary) was observed in the *Passiflora *group on day 30.

The severity of insomnia assessed using ISI was reduced significantly in the *Passiflora *group compared to the placebo group on day 15 and day 30. No significant difference was observed in the serum cortisol (morning) level between the two groups; however, a higher percentage (17.21%) reduction was observed in the *Passiflora *group compared to the 11.04% reduction in the placebo group. Serum cortisol levels remained within normal range in both groups both at baseline and at the end of the study.

A statistically significant reduction in fatigue was observed in the *Passiflora *group compared to the placebo group on day 30. Significantly lesser interference of sleep on daytime mood, ability to function at work, concentration, and memory was observed in the *Passiflora *group as compared to the placebo group on day 15 and day 30. None of the subjects from both groups required rescue medication (sedatives) during the study period. Significantly better quality of sleep as assessed using PSQI was observed in the *Passiflora *group compared to the placebo group on day 30.

In a Korean study, *Passiflora incarnata* increased the total sleep time in adults with insomnia in a prospective, double-blind, randomized, placebo-controlled study compared to the placebo [15]. However, other parameters like ISI, quality of sleep, sleep latency, total arousal, WASO, and stress did not show a significant difference between the groups probably because of the low dose (60 mg per day) and shorter duration (two weeks) of the treatment in the study [15]. In a clinical trial, 12 weeks of treatment with a dried ethanolic extract of passion flower showed significant improvements in stress resistance (resilience) and quality of life in patients suffering from nervous restlessness [16]. Passion flower has been used in the management of neurotic disorders, stress, anxiety, insomnia, opioid withdrawal symptoms, morphine dependence, convulsions, and neuralgia with low addiction potential [14-17]. Various research studies have shown that *Passiflora incarnata* is useful in the management of sleep disorders. Studies revealed that *Passiflora incarnata *modulates the gamma-aminobutyric acid (GABA) neurotransmission system and helps to improve sleep quality [14]. In an animal study, *Passiflora incarnata *modulated the aggressive and abnormal behaviors of animals and lowered the salivary cortisol level [17]. It has been shown that *Passiflora *reduced anxiety and improved spatial memory in rats indicating the involvement of GABAA receptors [18]. Like the Korean clinical study on *Passiflora incarnata* [15], this study also did not show any safety concerns or adverse events indicating the tolerability of the plant in the Indian population as well. One of the limitations of the study was that no sleep assessment instruments like actigraphy or ballistocardiography were used.

## Conclusions

Overall, we could say that the extract of *Passiflora incarnata* aerial parts could be used safely in the management of sleep and stress. This is because the extract improved total sleep time and reduced stress in Indian subjects with stress and sleep problems. It improved the quality of sleep and provided sound sleep with lesser disturbance. Also, it improved associated symptoms like mood, ability to work, concentration, and memory. All these effects were observed without any side effects or adverse events.
